# LIFE SATISFACTION MODERATES LIFESPAN DECLINES IN STRESS REACTIVITY

**DOI:** 10.1093/geroni/igae098.0555

**Published:** 2024-12-31

**Authors:** William Chopik

**Affiliations:** Michigan State University East Lansing, East Lansing, Michigan, United States

## Abstract

Positive psychological well-being is often conceptualized as a protective factor for maintaining health across the lifespan. But why does psychological well-being confer such benefits? One potential possibility is that it shapes how we respond to stressors on a daily basis. However, much of existing research on this topic will examine these associations in the context of short-term experience sampling studies. Although useful in linking well-being to how we perceive and experience stress, this research can be limited in that it rarely tests prospective effects of well-being on stress reactivity over longer intervals. In the current study, I examined changes in stress reactivity across three waves of the National Study of Daily Experiences from approximately 1996 to 2017 (N = 1,500; Mage = 46.74; 54.1% women; 91.7% white) as a function of life satisfaction at wave 1. Employing three-level multi-level models, I reproduced recent research showing stress reactivity declines across life (b = -.02, p <.001); life satisfaction predicts lower stress reactivity both overall (b = -.04, p <.001) and in the context of a higher number of average (b = -.04, p <.001) and daily stressors (b = -.02, p <.001). Integrating repeated measures of life satisfaction and stress reactivity in the context of a random-intercept cross-lagged panel model suggested that reduced stress reactivity might lead to increases in life satisfaction across life. The results will be discussed in the context of how daily processes might “trickle up” to cultivate variation in global reports of well-being.

